# Is the Immunopeptidome Getting Darker?: A Commentary on the Discussion around Mishto et al., 2019

**DOI:** 10.3389/fimmu.2021.720811

**Published:** 2021-07-13

**Authors:** Anthony W. Purcell

**Affiliations:** Department of Biochemistry and Molecular Biology, and Infection and Immunity Program, Biomedicine Discovery Institute, Monash University, Clayton, VIC, Australia

**Keywords:** HLA, mass spectrometry - LC-MS/MS, immunopeptidome, proteasome, spliced peptides

## Introduction

The immune system is perhaps the most ancient practitioner of proteomics; and as we delve into the more nuanced aspects of antigen processing and presentation, we learn more of the complexity of this essential process and Into a debate amongst the human practitioners of immunopeptidomics threatens to boil over and confer reputational damage to both the field and the investigators who use this technique.

Traditionally the art of proteomics has used the identification of tryptic peptides to infer protein expression, a process termed “bottom-up” proteomics. Notably, an analogous process is used by antigen presenting cells to alert the immune system to the presence of infectious micro-organisms, malignancy and other abnormalities within the body. This process known as antigen processing and presentation relies on the selection of peptide fragments of antigens and their presentation on the surface of cells as a bound complex with Major Histocompatibility Complex (MHC) molecules. The isolation and characterisation of these MHC-bound peptides, most commonly through the use of liquid chromatography and mass spectrometry, is a rapidly growing field coined immunopeptidomics.

Immunopeptidomics as a field has rapidly matured in the past decade and has moved from the domain of just a handful of specialised laboratories to now being more generally applied by the proteomics and immunology communities. The genesis of immunopeptidomics can be traced back to early work of the groups of Don Hunt with Victor Englehard and Alessandro Sette (1–5) and Hans-Georg Rammensee (6–9). It was in the early 90’s that I joined the field and was immediately struck by the generosity and encouragement of the established investigators – sharing anecdotes, methods and solutions to problems with a focus on growing the discipline and answering key questions of immune specificity whilst untangling the complexity of antigen presentation. This is something I have never forgotten and it has inspired me to “give back” to the field happily training the numerous visitors to my laboratory and publishing our methodology widely and in great depth (10, 11).

Fast forwarding a few decades there have been drastic and exciting changes to the immunopeptidomics field. Improvements in instrumentation, nanoscale separation sciences and data acquisition have provided extremely rich datasets. The maturation of informatic approaches to handle this challenging data has also facilitated routine analysis of these non-tryptic peptides allowing thousands of confident identifications to be made. However, it is clear that there are still informatic challenges in the peptide-centric data analysis pipelines (12, 13) with a much lower proportion of spectra confidently assigned from immunopeptidomics data compared with more conventional samples such as tryptic digests of cell lysates. The search to explain these dark elements of the immunopeptidome has driven differences in interpretation and peptide sequence assignments to these MS/MS spectra (12, 14–25).

These issues are most apparent when it comes to the presence of post-translationally spliced peptides in the immunopeptidome. Their presence in the immunopeptidome first came to note with pioneering work from Jonathan Yewdell and James Yang (26) and Nathalie Vigneron and Benoit van den Eynde (27–31) linking these peptides with immune responses primarily in cancer, as also confirmed by others (21, 32). Conventional wisdom immediately decreed their existence and the proteasomal generation of such spliced peptides as being a rare event. Their presence in several other disease states has recently been reviewed (23, 33) but perhaps most surprisingly (and controversially) the frequency of these peptides in the HLA class I immunopeptidome was reported to be up to one third of all peptides identified isolated HLA peptide ligands (15). This ground-breaking study from Liepe and colleagues (15, 19) has been confirmed by some (16) and disputed by others (17). But what are the outcomes from this study and the subsequent response by the field? It has certainly shone a spotlight on the uncomfortably large proportion of peptides in the immunopeptidome that cannot be readily assigned using reference proteomes. It has also driven developments in the informatics used to search immunopeptidomics data with improvements in assignments of post-translational modifications (34, 35), incorporation of proteogenomics databases (36) and a series of alternative workflows to address the presence of spliced peptides (16, 18, 20–22, 24, 25, 37–42). However, no consensus has been reached with somewhere between 0 and 45% of peptides in the immunopeptidome believed to be spliced. As a field where we go from here is critical – we could polarize to one extreme or the other, sit on the fence or actually address the question of what is the source of these peptide antigens that comprise the dark immunopeptidome. It is clear that several possibilities are emerging to explain a proportion of these peptides (in addition to a proportion of spliced peptides) including unanticipated post-translational modifications (24); the presence of poorly annotated non-canonical sources of antigen such as small open reading frames, translated 5′ UTRs, exon–exon junctions, intronic regions, non-canonical reading frames or antisense transcripts all of which can potentially generate immunogenic non-canonical peptides (36, 43); endogenous retroviral elements (44), the presence of microbial peptides (45). Each source of non-canonical peptide will have its supporters and detractors but perhaps none are essentially wrong and the major limitation at present is the ability to differentiate between the various explanations for the experimentally acquired MS/MS data we generate?

It is also time as a field that we decide how we debate these issues and in my opinion time to stop subjectively re-searching others data but to design and implement our own experiments to more definitely corroborate or repudiate various sources of antigenic peptides in the immunopeptidome. Our field has made great strides and can look back at great community initiatives: the human immunopeptidome project (46) that strives to advance the documentation of peptides available for immune recognition in a variety of healthy and diseased states; minimal information requirements for an immunopeptidomics experiment (47) and HLA peptide atlases (48, 49) and data repositories (50) that have driven improvements in predictive algorithms and will provide the data required for the next generation of AI-based informatic tools (25). Let’s use this same spirit to explore the immunopeptidome and illuminate its dark side as a community united in this purpose.

## Author Contributions

AWP wrote this commentary.

## Conflict of Interest

The author declares that the research was conducted in the absence of any commercial or financial relationships that could be construed as a potential conflict of interest.

## References

[1] Hunt DF Michel H Dickinson TA Shabanowitz J Cox AL Sakaguchi K. Peptides Presented to the Immune System by the Murine Class II Major Histocompatibility Complex Molecule I-A^d^ . Science (1992) 256:1817–20. 10.1126/science.1319610 1319610

[2] Henderson RAM H Sakaguchi K Shabaniwitz J Appela E Hunt DF Engelhard VH. HLA-A2.1-Associated Peptides From a Mutant Cell Line: A Second Pathway of Antigen Presentation. Science (1992) 255:1264–6. 10.1126/science.1546329 1546329

[3] Hunt DF Henderson RA Shabanowitz J Sacaguchi K Michel H Sevilir N. Characterization of Peptides Bound to the Class I MHC Molecule HLA-A2.1 by Mass Spectrometry. Science (1992) 255:1261–3. 10.1126/science.1546328 1546328

[4] Sette A Ceman S Kubo RT Sakaguchi K Appella E Hunt DF. Invariant Chain Peptides in Most HLA-DR Molecules of an Antigen- Processing Mutant. Science (1992) 258(5089):1801–4. 10.1126/science.1465617 1465617

[5] Henderson RA Cox AL Sakaguchi K Appella E Shabanowitz J Hunt DF. Direct Identification of an Endogenous Peptide Recognized by Multiple HLA-A2.1-Specific Cytotoxic T Cells. Proc Natl Acad Sci USA (1993) 90(21):10275–9. 10.1073/pnas.90.21.10275 PMC47757 7694286

[6] Rotzschke O Falk K Deres K Schild H Norda M Metzger J. Isolation and Analysis of Naturally Processed Viral Peptides as Recognized by Cytotoxic T Cells. Nature (1990) 348(6298):252–4. 10.1038/348252a0 1700304

[7] Falk K Rötzschke O Deres K Metzger J Jung G Rammensee H-G. Identification of Naturally Processed Viral Nonapeptides Allows Their Quantification in Infected Cells and Suggests an Allele-Specific T Cell Epitope Forecast. J Exp Med (1991) 174:425–34. 10.1084/jem.174.2.425 PMC2118916 1713253

[8] Falk K Rötzschke O Stevanovic S Jung G Rammensee H-G. Allele-Specific Motifs Revealed by Sequencing of Self-Peptides Eluted From MHC Molecules. Nature (1991) 351:290–6. 10.1038/351290a0 1709722

[9] Rammensee HG Falk K Rotzschke O. Peptides Naturally Presented by MHC Class I Molecules. Annu Rev Immunol (1993) 11:213–44. 10.1146/annurev.iy.11.040193.001241 8476560

[10] Pandey K Ramarathinam SH Purcell AW. Isolation of HLA Bound Peptides by Immunoaffinity Capture and Identification by Mass Spectrometry. Curr Protoc (2021) 1(3):e92. 10.1002/cpz1.92 33769717

[11] Purcell AW Ramarathinam SH Ternette N. Mass Spectrometry-Based Identification of MHC-Bound Peptides for Immunopeptidomics. Nat Protoc (2019) 14(6):1687–707. 10.1038/s41596-019-0133-y 31092913

[12] Admon A. Are There Indeed Spliced Peptides in the Immunopeptidome? Mol Cell Proteomics (2021) 100099. 10.1016/j.mcpro.2021.100099 PMC8724635 34022431

[13] Faridi P Purcell A Croft N. In Immunopeptidomics We Need a Sniper Instead of a Shotgun. Proteomics (2018) 18(12):7. 10.1002/pmic.201700464 29377634

[14] Haen SP Löffler MW Rammensee H-G Brossart P. Towards New Horizons: Characterization, Classification and Implications of the Tumour Antigenic Repertoire. Nat Rev Clin Oncol (2020) 17(10):595–610. 10.1038/s41571-020-0387-x 32572208 PMC7306938

[15] Liepe J Marino F Sidney J Jeko A Bunting DE Sette A. A Large Fraction of HLA Class I Ligands Are Proteasome-Generated Spliced Peptides. Science (2016) 354(6310):354–8. 10.1126/science.aaf4384 27846572

[16] Faridi P Li C Ramarathinam SH Vivian JP Illing PT Mifsud NA. A Subset of HLA-I Peptides Are Not Genomically Templated: Evidence for Cis- and Trans-Spliced Peptide Ligands. Sci Immunol (2018) 3(28):eaar3947. 10.1126/sciimmunol.aar3947 30315122

[17] Mylonas R Beer I Iseli C Chong C Pak HS Gfeller D. Estimating the Contribution of Proteasomal Spliced Peptides to the HLA-I Ligandome. Mol Cell Proteomics (2018) 17(12):2347–57. 10.1074/mcp.RA118.000877 PMC6283289 30171158

[18] Rolfs Z Solntsev SK Shortreed MR Frey BL Smith LM. Global Identification of Post-Translationally Spliced Peptides With Neo-Fusion. J Proteome Res (2018) 18(1):349–58. 10.1021/acs.jproteome.8b00651 PMC6465104 30346791

[19] Liepe J Sidney J Lorenz FKM Sette A Mishto M. Mapping the MHC Class I-Spliced Immunopeptidome of Cancer Cells. Cancer Immunol Res (2019) 7(1):62–76. 10.1158/2326-6066.CIR-18-0424 30425108 PMC12032831

[20] Erhard F Dölken L Schilling B Schlosser A. Identification of the Cryptic HLA-I Immunopeptidome. Cancer Immunol Res (2020) 8(8):1018–26. 10.1158/2326-6066.CIR-19-0886 32561536

[21] Faridi P Woods K Ostrouska S Deceneux C Aranha R Duscharla D. Spliced Peptides and Cytokine-Driven Changes in the Immunopeptidome of Melanoma. Cancer Immunol Res (2020) 8(10):1322–34. 10.1158/2326-6066.CIR-19-0894 32938616

[22] Mishto M. What We See, What We Do Not See, and What We do Not Want to See in HLA Class I Immunopeptidomes. Proteomics (2020) 12:e2000112. 10.1002/pmic.202000112 32533627

[23] Faridi P Dorvash M Purcell AW. Spliced HLA-Bound Peptides: A Black Swan Event in Immunology. Clin Exp Immunol (2021) 204(2):179–88. 10.1111/cei.13589 PMC8062993 33644851

[24] Lichti CF. Identification of Spliced Peptides in Pancreatic Islets Uncovers Errors Leading to False Assignments. Proteomics (2021) 21(7-8):e2000176. 10.1002/pmic.202000176 33548107

[25] Wilhelm M Zolg DP Graber M Gessulat S Schmidt T Schnatbaum K. Deep Learning Boosts Sensitivity of Mass Spectrometry-Based Immunopeptidomics. Nat Commun (2021) 12(1):3346. 10.1038/s41467-021-23713-9 34099720 PMC8184761

[26] Hanada K-I Yewdell JW Yang JC. Immune Recognition of a Human Renal Cancer Antigen Through Post-Translational Protein Splicing. Nature (2004) 427(6971):252–6. 10.1038/nature02240 14724640

[27] Vigneron N Stroobant V Chapiro J Ooms A Degiovanni G Morel S. An Antigenic Peptide Produced by Peptide Splicing in the Proteasome. Science (2004) 304(5670):587–90. 10.1126/science.1095522 15001714

[28] Dalet A Vigneron N Stroobant V Hanada K Van den Eynde BJ. Splicing of Distant Peptide Fragments Occurs in the Proteasome by Transpeptidation and Produces the Spliced Antigenic Peptide Derived From Fibroblast Growth Factor-5. J Immunol (Baltimore Md: 1950) (2010) 184(6):3016–24. 10.4049/jimmunol.0901277 20154207

[29] Dalet A Robbins PF Stroobant V Vigneron N Li YF El-Gamil M. An Antigenic Peptide Produced by Reverse Splicing and Double Asparagine Deamidation. Proc Natl Acad Sci USA (2011) 108(29):E323–31. 10.1073/pnas.1101892108 PMC3142003 21670269

[30] Michaux A Larrieu P Stroobant V Fonteneau J-F Jotereau F Van den Eynde BJ. A Spliced Antigenic Peptide Comprising a Single Spliced Amino Acid Is Produced in the Proteasome by Reverse Splicing of a Longer Peptide Fragment Followed by Trimming. J Immunol (2014) 192(4):1962–71. 10.4049/jimmunol.1302032 24453253

[31] Vigneron N Ferrari V Stroobant V Abi Habib J Van den Eynde BJ. Peptide Splicing by the Proteasome. J Biol Chem (2017) 292(51):21170–9. 10.1074/jbc.R117.807560 PMC5743089 29109146

[32] Ebstein F Textoris-Taube K Keller C Golnik R Vigneron N Van den Eynde BJ. Proteasomes Generate Spliced Epitopes by Two Different Mechanisms and as Efficiently as Non-Spliced Epitopes. Sci Rep (2016) 6(1):24032. 10.1038/srep24032 27049119 PMC4822137

[33] Liepe J Ovaa H Mishto M. Why do Proteases Mess Up With Antigen Presentation by Re-Shuffling Antigen Sequences? Curr Opin Immunol (2018) 52:81–6. 10.1016/j.coi.2018.04.016 29723668

[34] Kong AT Leprevost FV Avtonomov DM Mellacheruvu D Nesvizhskii AI. Msfragger: Ultrafast and Comprehensive Peptide Identification in Mass Spectrometry-Based Proteomics. Nat Methods (2017) 14(5):513–20. 10.1038/nmeth.4256 PMC5409104 28394336

[35] Yu F Teo GC Kong AT Haynes SE Avtonomov DM Geiszler DJ. Identification of Modified Peptides Using Localization-Aware Open Search. Nat Commun (2020) 11(1):4065. 10.1038/s41467-020-17921-y 32792501 PMC7426425

[36] Ruiz Cuevas MV Hardy MP Hollý J Bonneil É Durette C Courcelles M. Most non-Canonical Proteins Uniquely Populate the Proteome or Immunopeptidome. Cell Rep (2021) 34(10):108815. 10.1016/j.celrep.2021.108815 33691108 PMC8040094

[37] Paes W Leonov G Partridge T Nicastri A Ternette N Borrow P. Elucidation of the Signatures of Proteasome-Catalyzed Peptide Splicing. Front Immunol (2020) 11:563800. 10.3389/fimmu.2020.563800 33072102 PMC7541919

[38] Azoury ME Tarayrah M Afonso G Pais A Colli ML Maillard C. Peptides Derived From Insulin Granule Proteins Are Targeted by CD8(+) T Cells Across MHC Class I Restrictions in Humans and NOD Mice. Diabetes (2020) 69(12):2678–90. 10.2337/db20-0013 32928873

[39] Rolfs Z Müller M Shortreed MR Smith LM Bassani-Sternberg M. Comment on “A Subset of HLA-I Peptides Are Not Genomically Templated: Evidence for Cis- and Trans-Spliced Peptide Ligands”. Sci Immunol (2019) 4(38):eaaw1622. 10.1126/sciimmunol.aaw1622 31420320 PMC7039048

[40] Paes W Leonov G Partridge T Chikata T Murakoshi H Frangou A. Contribution of Proteasome-Catalyzed Peptide Cis-Splicing to Viral Targeting by CD8(+) T Cells in HIV-1 Infection. Proc Natl Acad Sci USA (2019) 116(49):24748–59. 10.1073/pnas.1911622116 PMC6900506 31748275

[41] Mishto M Mansurkhodzhaev A Ying G Bitra A Cordfunke RA Henze S. An in Silico-*In Vitro* Pipeline Identifying an HLA-a(*)02:01(+) KRAS G12V(+) Spliced Epitope Candidate for a Broad Tumor-Immune Response in Cancer Patients. Front Immunol (2019) 10:2572. 10.3389/fimmu.2019.02572 31803176 PMC6872521

[42] Faridi P Li C Ramarathinam SH Illing PT Mifsud NA Ayala R. Response to Comment on “A Subset of HLA-I Peptides Are Not Genomically Templated: Evidence for Cis- and Trans-Spliced Peptide Ligands”. Sci Immunol (2019) 4(38):eaaw8457. 10.1126/sciimmunol.aaw8457 31420321

[43] Starck SR Shastri N. Nowhere to Hide: Unconventional Translation Yields Cryptic Peptides for Immune Surveillance. Immunol Rev (2016) 272(1):8–16. 10.1111/imr.12434 27319338 PMC4916849

[44] Attig J Young GR Hosie L Perkins D Encheva-Yokoya V Stoye JP. LTR Retroelement Expansion of the Human Cancer Transcriptome and Immunopeptidome Revealed by *De Novo* Transcript Assembly. Genome Res (2019) 29(10):1578–90. 10.1101/gr.248922.119 PMC6771403 31537638

[45] Kalaora S Nagler A Nejman D Alon M Barbolin C Barnea E. Identification of Bacteria-Derived HLA-Bound Peptides in Melanoma. Nature (2021) 592(7852):138–43. 10.1038/s41586-021-03368-8 PMC9717498 33731925

[46] Vizcaíno JA Kubiniok P Kovalchik KA Ma Q Duquette JD Mongrain I. The Human Immunopeptidome Project: A Roadmap to Predict and Treat Immune Diseases. Mol Cell Proteomics (2020) 19(1):31–49. 10.1074/mcp.R119.001743 31744855 PMC6944237

[47] Lill JR van Veelen PA Tenzer S Admon A Caron E Elias J. Minimal Information About an Immuno-Peptidomics Experiment (MIAIPE). Proteomics (2018) 18(12):e1800110. 10.1002/pmic.201800110 29791771 PMC6033177

[48] Shao W Pedrioli PGA Wolski W Scurtescu C Schmid E Vizcaino JA. The Systemhc Atlas Project. Nucleic Acids Res (2018) 46(D1):D1237–47. 10.1093/nar/gkx664 PMC5753376 28985418

[49] Marcu A Bichmann L Kuchenbecker L Kowalewski DJ Freudenmann LK Backert L. HLA Ligand Atlas: A Benign Reference of HLA-Presented Peptides to Improve T-Cell-Based Cancer Immunotherapy. J Immunother Cancer (2021) 9(4):e002071. 10.1136/jitc-2020-002071 33858848 PMC8054196

[50] Martini S Nielsen M Peters B Sette A. The Immune Epitope Database and Analysis Resource Program 2003-2018: Reflections and Outlook. Immunogenetics (2020) 72(1–2):57–76. 10.1007/s00251-019-01137-6 31761977 PMC6970984

